# Improving survey methods in sero-epidemiological studies of injecting drug users: a case example of two cross sectional surveys in Serbia and Montenegro

**DOI:** 10.1186/1471-2334-9-14

**Published:** 2009-02-09

**Authors:** Ali Judd, Tim Rhodes, Lisa G Johnston, Lucy Platt, Violeta Andjelkovic, Danijela Simić, Boban Mugosa, Milena Simić, Sonja Žerjav, Ruth P Parry, John V Parry

**Affiliations:** 1 MRC Clinical Trials Unit, London, UK; 2 Centre for Research on Drugs and Health Behaviour, London School of Hygiene and Tropical Medicine, London, UK; 3 Tulane University, School of Public Health and Tropical Medicine, New Orleans, Louisiana, USA; 4 International Aid Network, Belgrade, Serbia; 5 Institute of Public Health of Serbia, Belgrade, Serbia; 6 Institute of Public Health, Podgorica, Montenegro; 7 Department of Social Medicine, University of Bristol, Bristol, UK; 8 HIV/AIDS Centre, Institute for Infectious and Tropical Diseases, School of Medicine, University of Belgrade, Serbia; 9 Virus Reference Department, Health Protection Agency Centre for Infections, London, UK

## Abstract

**Background:**

Little is known about the prevalence of HIV or HCV in injecting drug users (IDUs) in Serbia and Montenegro. We measured prevalence of antibodies to HIV (anti-HIV) and hepatitis C virus (anti-HCV), and risk factors for anti-HCV, in community-recruited IDUs in Belgrade and Podgorica, and determined the performance of a parallel rapid HIV testing algorithm.

**Methods:**

Respondent driven sampling and audio-computer assisted survey interviewing (ACASI) methods were employed. Dried blood spots were collected for unlinked anonymous antibody testing. Belgrade IDUs were offered voluntary confidential rapid HIV testing using a parallel testing algorithm, the performance of which was compared with standard laboratory tests. Predictors of anti-HCV positivity and the diagnostic accuracy of the rapid HIV test algorithm were calculated.

**Results:**

Overall population prevalence of anti-HIV and anti-HCV in IDUs were 3% and 63% respectively in Belgrade (n = 433) and 0% and 22% in Podgorica (n = 328). Around a quarter of IDUs in each city had injected with used needles and syringes in the last four weeks. In both cities anti-HCV positivity was associated with increasing number of years injecting (eg Belgrade adjusted odds ratio (AOR) 5.6 (95% CI 3.2–9.7) and Podgorica AOR 2.5 (1.3–5.1) for ≥ 10 years v 0–4 years), daily injecting (Belgrade AOR 1.6 (1.0–2.7), Podgorica AOR 2.1 (1.3–5.1)), and having ever shared used needles/syringes (Belgrade AOR 2.3 (1.0–5.4), Podgorica AOR 1.9 (1.4–2.6)). Half (47%) of Belgrade participants accepted rapid HIV testing, and there was complete concordance between rapid test results and subsequent confirmatory laboratory tests (sensitivity 100% (95%CI 59%–100%), specificity 100% (95%CI 98%–100%)).

**Conclusion:**

The combination of community recruitment, ACASI, rapid testing and a linked diagnostic accuracy study provide enhanced methods for conducting blood borne virus sero-prevalence studies in IDUs. The relatively high uptake of rapid testing suggests that introducing this method in community settings could increase the number of people tested in high risk populations. The high prevalence of HCV and relatively high prevalence of injecting risk behaviour indicate that further HIV transmission is likely in IDUs in both cities. Urgent scale up of HIV prevention interventions is needed.

## Background

While an evidence base characterising the epidemiology of HIV and hepatitis C virus (HCV) among injecting drug users (IDUs) has emerged in many Eastern Europe countries,[1,2] less is known of South Eastern European countries, including the Western Balkans. This is despite countries in the Western Balkans and Eastern Europe sharing many characteristics of the HIV risk environment, such as major social and economic transition linking with expanding illicit drug markets, increased poverty, and weakening public health infrastructure.[3] Studies suggest low prevalence of HIV in IDUs in the region but higher prevalence of HCV, with estimates ≥ 50% in Bulgaria, Romania, Slovenia and Croatia.[4] There are only two published surveys of HIV prevalence among IDUs in Serbia,[5,6] and none in Montenegro. In Belgrade, HIV prevalence was 39% in 472 IDUs admitted to a drug treatment centre in 1987/8.[5] A later study of IDUs treated for drug problems in Belgrade found that 44% were anti-HIV positive.[6] HIV and AIDS registry data suggest that injecting drug use is yet to be a major transmission route for HIV in Serbia and Montenegro; between 2002 and 2006, 494 newly diagnosed HIV infections were reported in Serbia, of which 66 were attributed to injecting drug use, and similarly 25 in Montenegro, of which 3 were in IDUs.[7] No estimates of anti-HCV prevalence in IDUs in Serbia and Montenegro have been published.

A number of contextual factors shape the delivery of HIV and HCV testing services for IDUs in Serbia and Montenegro, which in turn introduce challenges for conducting sero-epidemiological research.[8,9] Resources for the delivery of low-threshold and confidential HIV and HCV antibody testing at testing centres is limited, with reports of inconsistent availability of testing kits. Qualitative research suggests reluctance among IDUs to access local testing centres, related to concerns over confidentiality, a lack of trust in services and expertise, and experience of stigma when interfacing with health care and other institutions.[8,9] Avoidance of service contact can be a strategy of stigma avoidance.[8] There exists a culture of silence and non-disclosure regarding viral infections among some sectors of the IDU population.[8] This emphasizes the need for sero-epidemiological research approaches which focus upon methods of community recruitment. It also emphasizes the potential afforded by delivering HIV and other testing services directly in the community and outside the laboratory. At the time of the study, however, all HIV and HCV testing services in Serbia and Montenegro were provided within designated medical centres. This study obtained specific approval by the relevant Government Ministries to offer point-of-care tests (POCT) for HIV directly in community settings as part of its aim to explore improvements in survey methods in sero-epidemiological research in hidden populations of IDUs.

### Sampling hidden populations

Although most epidemiological studies measuring HIV and HCV prevalence in IDUs recruit a theoretically biased selection of participants from drug treatment clinics, some have used "snowball sampling", often with peer interviewers, to recruit IDUs from non-treatment community settings. These "community based" snowball samples are often themselves biased by several factors, including oversampling of participants with larger social networks, insufficient recruitment waves rendering a final sample with characteristics biased by the initial participants, and widespread masking (protecting friends due to privacy concerns) and volunteerism.[10,11] Recently, a modified version of snowball sampling, known as "respondent driven sampling" (RDS), has been developed which aims to eliminate several such biases to achieve representative samples of high risk, hard-to-react populations.[10,12] RDS uses a coupon system of recruitment whereby peers recruit their peers to create numerous recruitment waves, long recruitment chains and an incentive mechanism for participating in the study; this in turn helps eliminate some of the biases inherent in snowball sampling methods.[10,13] Furthermore, data gathered using RDS are subject to specialised statistical analysis to mitigate factors associated with biased recruitment.[13,14] However, the method has been associated with operational, design and analytical challenges in some studies [15] and there is some discussion about the ethics of incentive-based recruitment systems [16]. RDS requires careful implementation to maximise its success.

### Services at the point of recruitment

RDS studies usually have fixed locations where participants redeem their coupons and enrol in the survey. This offers researchers the opportunity to provide much needed services to these populations, as well as improve the validity and reliability of survey methods. For instance, many previous studies have employed unlinked anonymous testing of biological samples in which, for practical reasons, results are not returned to participants: turnaround times for returning results from a reference laboratory are usually in the order of days rather than hours; re-contacting marginalised groups to deliver test results are poor.[17] Point-of-care tests (POCT) for HIV offer a novel and alternative strategy whereby the testing is moved from the laboratory into the community, in this case to the RDS study site, and results can be provided to the most hard-to-reach populations at the same visit.[18] POCTs are simple to use and thus facilitate administration by non-laboratory staff who have received appropriate training and had their competency checked. This enables voluntary counselling and testing (VCT) programmes to be extended to community settings and offered as an added incentive to participate in epidemiological studies. Fixed sites also enable studies to use audio-computer assisted survey interviewing (ACASI), which has been shown to elicit higher reports of high risk and often stigmatised behaviours, which are assumed to be closer to true levels.[19,20]

Recent studies suggest that rapid HIV testing in community settings can effectively target groups at high risk of HIV infection.[21] Rapid testing has been conducted in parks, shelters, syringe exchanges, community clinics, VCT programmes and antenatal clinics. [21-25] The performance of algorithms employing several different POCTs either in consecutive or parallel testing strategies has also been reported, with varying outcomes. One field evaluation suggested that different combinations of POCTs provided sensitivity and specificity similar to standard enzyme immunoassays and Western Blot.[22] Another study evaluating the performance of the Determine HIV-1/2 in pregnant women in Mexico reported high sensitivity and specificity, though a lower positive predictive value associated with the low prevalence of anti-HIV in this population.[25] Another study reported successful implementation of rapid HIV testing in several different types of community settings but only measured the positive predictive value for those receiving confirmatory tests after testing anti-HIV positive on the rapid HIV assay.[21] A study employing the Determine and Uni-Gold rapid HIV test assays in Uganda reported good sensitivity, though specificity was compromised because of the occurrence of weak reactions with the test band. These weak reactions were associated in the main with the Determine test, most of which were later ascertained to be HIV negative on enzyme immunoassay (EIA) and Western blotting.[26]

This paper reports on the findings of two parallel surveys of IDUs conducted in Belgrade and Podgorica in 2005 using RDS. Both recruited IDUs from community settings and employed ACASI, with the Belgrade survey including a voluntary confidential rapid HIV testing component and a diagnostic accuracy study.

## Methods

Two cross sectional, anonymous, surveys of IDUs were undertaken simultaneously in Belgrade and Podgorica in September and October 2005. Both surveys used identical sampling and interview methods. Inclusion criteria were: injecting drugs in the previous four weeks; living or working in Belgrade or Podgorica; aged 18 years or older; willing to give a dried blood spot (DBS) for antibody testing; and not having been interviewed for the study previously.

Participants were recruited using RDS,[10] and were interviewed at fixed sites located in the centre of each city. The Belgrade site was housed within a discrete building used by the International AID Network (IAN), an NGO working with vulnerable people in South Eastern Europe. The Podgorica site was housed within some unmarked rooms in a mini shopping mall near the centre of the city, and comprised part of the premises of a local NGO called "Juventas", which provides youth services in the city. Survey staff members in both cities included outreach workers and former injectors whom had extensive experience working with vulnerable populations in Serbia and Montenegro. Potential participants were screened using a list of questions about injecting drug preparation, type and cost and by looking at injection stigmata. Pre-survey training in both cities included RDS methodology, processing potential participants through screening for inclusion criteria, gathering RDS specific data for analysis, providing assistance to participants using ACASI, and managing incentive payments. Recruitment began with a set of initial recruits ("seeds") who met the inclusion criteria and who were diverse in terms of key characteristics of the drug injecting community (eg. age, gender, area of residence, duration of injecting).

After giving written informed consent, all participants were interviewed using ACASI, taking approximately 30 minutes to complete. The questionnaire included sections on socio-demographics, drug use, injecting and sexual risk behaviour, sex work, contact with the police, and HIV testing history. It was developed from other survey questionnaires[2,27], underwent piloting, and was informed by a linked qualitative study.[9]

Upon completion of the ACASI, all participants gave at least two DBS specimens for unlinked anonymous testing for anti-HIV and anti-HCV. Participants were given specific information detailing the unlinked anonymous nature of DBS testing in the Information Sheet for the study, and had the option to withdraw consent and opt out of the study. No identifiers were collected and so it was not possible to trace participants to report to them their test results. However all participants in Belgrade were offered voluntary anonymous rapid HIV testing and results, along with full pre- and post-test counselling. HIV test results were available within approximately 30 minutes after receiving pre-test counselling. Those testing HIV positive were referred to the Belgrade AIDS centre for appropriate confirmatory testing. HIV/AIDS educational materials, condoms and trained VCT counsellors were available throughout the duration of the study. In Podgorica, instead of rapid testing, participants were referred to a local, newly opened, free of charge HIV VCT centre.

IDUs received "primary" and "secondary" cash reimbursements of €10 each for participation in the survey and €5 for each of their recruited peers (maximum three) who met the inclusion criteria and enrolled in the survey.

### Laboratory methods – unlinked anonymous testing

Capillary blood was collected onto absorbent paper (Whatman 903, Astron, Huntingdon, UK) by finger prick using single use disposable lancets, and left to dry, thus becoming a DBS. Specimens were stored in plastic pouches with desiccant in on-site freezers, and were later transferred to the Clinic for Infectious and Tropical Diseases, Belgrade where they were stored at -25°C until testing.

DBS specimens were tested at the Clinic for Infectious and Tropical Diseases, Belgrade, under the supervision of staff of the Health Protection Agency Virus Reference Department. Six mm diameter disks were punched and each was placed in a designated well of a 96-well microplate. Plasma proteins, including antibodies, were eluted from the disks by overnight immersion in 200 μL of PBS/tween buffer.

#### Anti-HCV test

Testing for anti-HCV was carried out using a modified protocol for the Ortho HCV 3.0 SAVe ELISA (product number 940982, Ortho Diagnostics, Amersham).[28] The method employs a cut-off (CO) optical density (OD) of 0.090, and any DBS sample giving an OD value between 0.080 and 2.500 was re-eluted from a new punch and re-tested in duplicate using the same assay. Those that were repeatedly reactive, as well as those whose initial screening OD value exceeded the maximum for the plate reader (2.500), were considered to be anti-HCV positive. It was decided not to apply a second line assay with the intent to confirm the presence of anti-HCV primarily because the method employed is highly reproducible and specific. Even for a low HCV prevalence in IDUs of, for example, 30% the positive predictive value is 1.0.[28] Particularly with the added safeguard of duplicate re-testing of any specimen with an OD in the range 0.08 – 2.5 the application of a second test would not have added significantly to testing accuracy.

#### Anti-HIV test

For Anti-HIV testing an in-house version of the Abbott/Murex GACELISA HIV 1+2 enzyme immunoassay was employed.[29] The commercial GACELISA HIV 1+2 test was established in several studies to provide sensitivity and specificity close to 100% [30] and good sensitivity shortly after anti-HIV seroconversion.[31] Development of the commercial kit was led by one of the authors (JVP), but ceased to be manufactured in 2003. Consequently, an in-house version of the GACELISA was developed and validated at the HPA Centre for Infections. In brief, the assay employs a 96-well U-bottomed microplate solid phase (Nunc Maxisorp) coated with anti-human IgG antibodies (A0423, Dako UK Ltd), negative and positive controls (HIV-1 × 2 and HIV-2 × 1), specimen diluent (PBS with 10% fœtal bovine serum and 0.1% tween 20), each prepared in bulk at HPA, along with the conjugate (HRPO-tagged HIV-1 and HIV-2 antigens) and substrate (TMB), with their associated buffers scavenged from the Abbott/Murex HIV-1.2.0 GE95 EIA kit. The test is processed employing standard EIA equipment and absorbances are measured at 450 nm employing 630 nm as a reference. The cut-off is calculated as the mean OD of 4 negative controls + 0.2, and performance limits are applied that were established when the assay was developed and form part of routine QC monitoring. Validation of the assay was performed employing a panel of oral fluid specimens whose HIV status had been determined employing oral fluid specimens first characterised with the commercial Abbott Murex Diagnostics' GACELISA HIV 1+2 EIA (VK61). The validation panel comprised 194 anti-HIV-1 positive oral fluid specimens collected in the course of surveillance activities during 2001–2002 and 653 anti-HIV negative specimens collected during 2001 and 2003. The in-house GACELISA method detected 191/194 anti-HIV positive specimens, equivalent to a sensitivity of 99.5% (95% CI 96.7–100%). The falsely negative specimen was retested and found to be weakly reactive (OD/CO 1.21). Of 654 anti-HIV negative specimens all but 3 were non-reactive, a specificity of 99.5% (95% CI 98.5–99.9%). While all anti-HIV positive specimens were, by definition, detected by the commercial VK61 GACELISA HIV 1+2 EIA, it generated 7 false positive reactions, a specificity of 98.9% (95% CI 97.7–99.5%). Specimens that were anti-HIV reactive on initial testing were re-tested using the GACELISA. All repeatedly reactive samples and several of those that were reactive only on initial testing were examined by a modified Western Blot procedure (HIV Blot 2.2, Abbott Diagnostics, Maidenhead, UK).

For Anti-HIV testing an in-house version of the GACELISA HIV 1+2 enzyme immunoassay was employed [29] as the commercial kit is no longer manufactured. In local validation studies it had been shown to have an accuracy equivalent to that of the commercial GACELISA. Specimens that were anti-HIV reactive on initial testing were re-tested using the GACELISA. All repeatedly reactive samples and several of those that were reactive only on initial testing were examined by a modified Western Blot procedure (HIV Blot 2.2, Abbott Diagnostics, Maidenhead, UK).

### Rapid HIV testing

Rapid HIV testing was conducted using a parallel rapid testing algorithm. Capillary blood samples collected using single use disposable lancets were dropped into the sample ports of the rapid test devices according to the manufacturers' instructions. An additional DBS was taken from each participant for anti-HIV confirmatory testing (protocol as above) at a later date to estimate the diagnostic accuracy of the algorithm.

Two rapid HIV tests, Uni-Gold Recombinant HIV-1/2 (Trinity Biotech, Bray, Ireland) and Determine HIV-1/2/O (Abbott Laboratories, Abbott Park, IL, USA) were employed simultaneously to avoid the need to take a second blood sample should the first test be reactive. These tests have undergone evaluation by the World Health Organization; both had 100% sensitivity, with the Determine test having 99.4% and Uni-Gold 100% specificity.[32] A detailed protocol was followed which incorporated the manufacturers' instructions for use. Testing was conducted in a small temporary laboratory established within the Belgrade study site, and each test result was read by two trained technicians.

Additional file 1 describes the rapid testing algorithm. Reactivity of at least one rapid test result (two red bars, one in the "Test" and one in the "Control" window) indicated the possibility of HIV infection and the participant was referred to the Belgrade AIDS centre for appropriate confirmatory testing. Blood samples that were concordantly non-reactive, or where one test was non-reactive and the other invalid, were interpreted as indicating no evidence of HIV infection, and in which case no referral was made. In addition, the intensity of each reaction was recorded on a scale of one to four (no reaction; weak reaction; moderate reaction; strong reaction), as a quality check.

### Statistical analysis

Characteristics of the two samples of IDUs were compared, and the Respondent Driven Sampling Analysis Tool (RDSAT) v. 5.0.1 http://www.respondentdrivensampling.org was used to weight the sample to control for differences in network size, homophily (the tendency for people to preferentially recruit others similar to themselves) and recruitment patterns, to provide population based estimates and 95% confidence intervals (CI) of IDU characteristics in each city.[33] Correct implementation of RDS methodology (eg collection of participant's network sizes, and tracking who recruited whom) and analysis with RDSAT, provides representative estimates of the population sampled.[14]

We then evaluated predictors of anti-HCV positivity using univariable and multivariable logistic regression, considering the following variables: age group, sex and education; injecting drug use characteristics (duration of injecting, frequency of injecting, main drug injected, frequency of use of new needles/syringes, main source of new needles/syringes); injecting risk behaviours and ever having engaged in sex work; history of arrest, imprisonment, drug treatment and HCV testing. As a result of incorrect programming of ACASI, which allowed some questions to be skipped without a value being entered, several variables had ≥ 5% missing values: duration of injection (20%); frequency of injection (9%); main drug injected (9%); number of times using a needle/syringe before disposal (21%); main source of new needles/syringes (9%); ever injected with used needles/syringes (12%); injected with used needles/syringes in the last four weeks (19%); shared injecting paraphernalia (9%); ever engaged in sex work (16%); and ever detained or arrested by the police (36%). Therefore multiple imputations were used in multivariable models assuming the data were missing at random to avoid excluding different injectors from different analyses.[34] This involved creating or imputing plausible values for missing observations reflecting the uncertainty about the non-response model. Fifteen imputations were run on the missing data. The imputed datasets were combined and analysed together to allow for the uncertainty in the imputation model to be taken into account. Multivariable models were adjusted for robust standard errors as there was evidence of clustering by recruitment network. Statistical significance was assessed using Wald tests. All analyses were conducted using Stata 10 (Stata Corp, College Station, Texas).

Multivariable models were also adjusted for population weights on key demographic indicators to compensate for recruitment biases. Population weights adjusting for recruitment biases by education and sex were used for Belgrade, and age and education for Podgorica. Population weights were calculated using RDSAT v. 5.0.1 with anti-HCV status as the outcome variable.

### Ethics

Ethics approval was granted in the United Kingdom by the Charing Cross Research Ethics Committee. In Belgrade, the Ethics Committee of the Medical Faculty at the University of Belgrade approved the study and, in the absence of a suitable research ethics committee in Podgorica, approval for the study was granted by the Montenegrin Ministry of Health. In addition the Serbian Ministry of Health granted specific approval for the collection of biological specimens at study sites, and the rapid testing algorithm.

## Results

### Sample and population characteristics

Additional file 2 shows both sample and population characteristics of IDUs recruited in Belgrade (n = 433) and Podgorica (n = 328). After adjusting for differences in network sizes, homophily and recruitment patterns, most population estimates of most characteristics were similar to observed proportions in both cities. However, in Belgrade sample characteristics underestimated by ≥ 10% the proportion of IDUs in the population who had injected less than daily (47% observed v 59% population) and used a pharmacy for their main source of needles/syringes (72% v 83% respectively).

A quarter of the sample (27%) in Belgrade, and 41% in Podgorica, were aged <25 years; 22% and 7% respectively were female; 26% and 16% respectively reported having been in higher education (≥ 12 years schooling). A higher percentage of IDUs in Podgorica had shorter injecting careers (68% v 34% ≤ 4 years, respectively), obtained their syringes from an "other" source (29% v 1%), and had ever injected with used needles/syringes (66% v 50%) than those in Belgrade. Levels of other injecting and sexual risk behaviours were similar in both samples. Around a quarter in each city had injected with used needles and syringes in the last four weeks, and around 5% had ever engaged in sex work. A higher proportion of Belgrade injectors had been detained or arrested by the police in the last 12 months (62% v 52% respectively), imprisoned (43% v 34%), previously been in drug treatment (45% v 28%), and had ever been tested for HCV (51% v 24%).

### Laboratory testing for anti-HIV and anti-HCV

**Figure 1:**
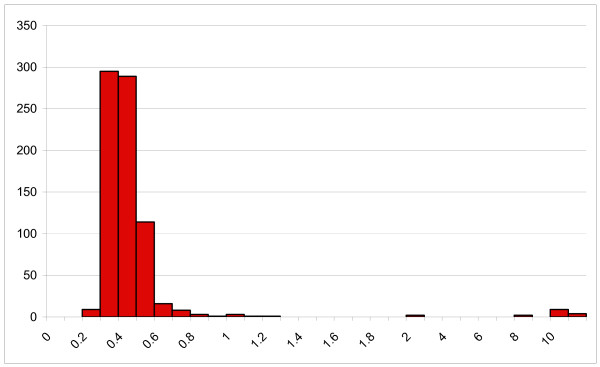
**GACELISA HIV 1+2 distribution of initial OD/CO values (n = 757)**.

DBS from the IDUs recruited in Belgrade and Podgorica were tested for anti-HIV and anti-HCV. Fifteen were initially reactive by GACELISA HIV 1+2, and there was excellent discrimination between non-reactive and reactive specimens (Figure 1). All 15 were repeatedly reactive (all OD/CO > 8.0), and all were confirmed to contain anti-HIV-1 by Western blot. A further seven specimens were initially weakly reactive (OD/CO 1.02–2.08), of which six were non-reactive on re-testing. As a quality check, three of these six were examined by Western Blot and were non-reactive. The remaining specimen (initial OD/CO 2.01) was not re-tested by GACELISA or Western blot and was therefore excluded from further analyses. Of the 315 DBS specimens tested in the diagnostic accuracy study 11 were initially reactive, ten of which were confirmed by Western blot to be anti-HIV-1 positive.

**Figure 2:**
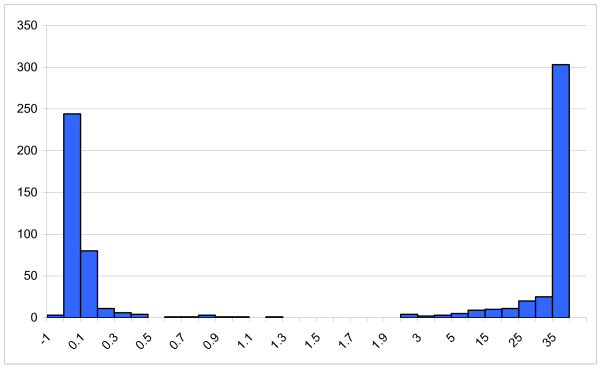
**Anti-HCV testing distribution of initial OD/CO values (n = 748)**.

**Figure 3:**
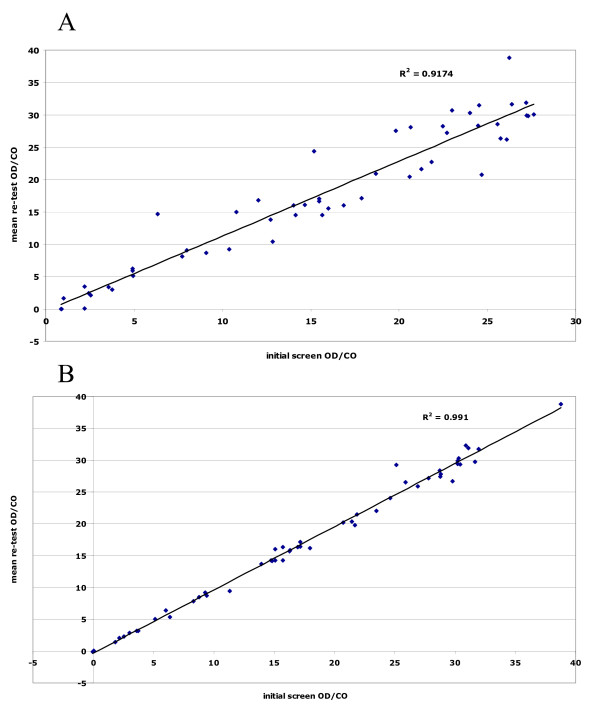
**A: Comparison of anti-HCV initial screen & mean re-test OD/CO values**. A new disc was punched eluted and tested in duplicate when the initial eluate gave an OD in the range 0.080 – 2.500 (n = 53). B: Comparison of duplicate anti-HCV OD/CO values on new eluates from DBS whose initial screen OD was in the range 0.080 – 2.500 (n = 53).

The anti-HCV reactivities also showed excellent polarisation between those non-reactive and those that were reactive (Figure 2). To check for reproducibility, 55 samples that gave an OD in the range 0.8–2.5 were retested in duplicate. All of those with an OD/CO > 1.0 were repeatedly reactive, apart from a single DBS whose initial OD/CO was weak, at 2.2 (retest OD/CO 0.03). The two samples with an initial OD/CO between 0.8 and 1.0 were non-reactive on repeat testing. The anti-HCV test method employed also exhibited very good quantitative reproducibility. Figure 3A illustrates the close correlation between the initial anti-HCV reactivity and the mean of the duplicate re-tests (R^2 ^= 0.92), and figure 3B that between the duplicate re-tests (R^2 ^= 0.99).

Overall adjusted population estimates of bloodborne virus prevalence were 3% (CI 2–6%) for anti-HIV and 63% (CI 57–70%) for anti-HCV for IDUs in Belgrade, and 0% (CI 0–1%) and 22% (CI 17–27%) for IDUs in Podgorica.

### Predictors of anti-HCV positivity

Additional file 3 displays univariable and multivariable predictors of anti-HCV positivity. Predictors of anti-HIV positivity were not explored as anti-HIV prevalence was too low. In Belgrade, females were more likely to be infected with HCV than males (adjusted odds ratio (AOR) 2.3, CI 1.5–3.7), and prevalence was lower in those with secondary or higher education compared to only primary (AOR 0.4, CI 0.2–0.8 and AOR 0.2, CI 0.1–0.6 respectively). Prevalence and odds also increased with length of injecting (eg. AOR 5.6, CI 3.2–9.7 for ≥ 10 years v 0–4 years), and were higher for those who injected daily compared to less than daily (AOR 1.6, CI 1.0–2.7). Those who used a needle or syringe two or more times prior to disposal had half the odds of anti-HCV compared to those using it just once (AOR 0.5, CI 0.3–0.7), whilst participants reporting having ever injected with used needles and syringes had twice the odds than those who had not (AOR 2.3, CI 1.0–5.4). Finally, for Belgrade anti-HCV prevalence was higher in those who had ever been in prison (AOR 1.6, CI 1.1–2.5).

In Podgorica, as in Belgrade, anti-HCV positivity was associated with longer lengths of injecting, albeit less markedly (AOR 2.5, CI 1.3–5.1 for ≥ 10 years v 0–4 years), as well as daily injecting (AOR 2.1, CI 1.3–5.1) and ever having injected with used needles and syringes (AOR 1.9, CI 1.4–2.6) (Additional file 3). In contrast to Belgrade, anti-HCV positivity in Podgorica was also raised in older participants (AOR 3.5, CI 2.2–5.8 for those aged ≥ 30 v <25 years), those who had been detained or arrested by the police in the last 12 months (AOR 2.5, CI 1.2–5.2) and those ever tested for HCV (AOR 2.0, CI 1.4–2.9).

There were minor differences in predictors of anti-HCV positivity in the multivariable imputed models displayed in Additional file 3 and ordinary multivariable models (not shown). For Belgrade, frequency of injection was associated with anti-HCV in the imputed but not the ordinary model (OR = 1.3, CI 0.8–2.21), as was ever injected with used needles/syringes (OR = 2.5, CI 0.9–7.1). For Podgorica higher education was associated with anti-HCV positivity in the ordinary model (OR = 6.9 CI 3.1–15.1) but not the imputed model, as was ever sex work (OR = 2.2, CI 1.0–4.5) and ever received drug treatment (OR = 3.3, CI 2.0–5.5 for current treatment).

### Rapid testing uptake and diagnostic accuracy

All participants in Belgrade were offered a rapid HIV test, of whom 204 (47%, CI 42%–52%) took up the offer. Five withdrew after pre-test counselling, resulting in 199 participants with rapid test results. For these, an additional DBS was collected from 176 participants for confirmatory testing.

Additional file 4 shows a comparison of the results from the rapid test algorithm and the confirmatory testing. There was complete concordance between the rapid test algorithm and confirmatory tests, resulting in excellent specificity and negative predictive value (100.0%, CI 97.8–100.0% for both indicators), as well as sensitivity and positive predictive value (100%, CI 59.0–100.0% for both indicators) albeit with a wide confidence interval reflecting the small number of true positives in the sample.

The intensity of reaction for all 191 negative results on the Unigold test was recorded as "0" (no reaction; for one participant the Determine test alone was employed). For the seven positive reactions, one scored "2" (moderate reaction) and six scored "3" (strong reaction). Similarly for the Determine, all 192 negative results scored "0", whilst all seven positive reactions scored "3".

## Discussion

A simple analysis of the laboratory findings indicated how well the test protocols developed for anti-HIV and anti-HCV testing of DBS segregate reactive and non-reactive specimens. Moreover, retesting the anti-HIV reactive specimens and confirmatory testing with Western blot highlighted the specificity of the GACELISA method. Similarly, retesting the minority of anti-HCV specimens whose reactivity was not at the extremes of the distribution of reactivities demonstrated the reproducibility of the method. Our original description of the anti-HCV DBS method indicated that it was highly accurate and these findings are consistent with that conclusion.[28]

### Prevalence and risk

In our study, prevalence of anti-HIV was 3% in Belgrade and 0% in Podgorica, suggesting that injecting drug use is yet to have a major impact on the HIV epidemic in either city. However, the much higher prevalence of anti-HCV, at around 60% for Belgrade and 20% for Podgorica, and high levels of injecting risk behaviour, indicate the need for a cautious response as levels of risk behaviour appear sufficient to enable considerable onward bloodborne virus transmission. Our study indicates that only half of IDUs in Belgrade and only a quarter of IDUs in Podgorica had ever been tested for HCV, and around a quarter of IDUs in both cities reported injecting with used needles and syringes in the previous four weeks.

IDUs in Podgorica were younger, had shorter duration of injecting, and were less likely to inject on a daily basis than their Belgrade counterparts, suggesting a more recent introduction of injecting drug use in Podgorica. Three risk factors for anti-HCV were the same for the two cities: anti-HCV positivity increased with longer lengths of injecting; it was higher for daily injectors; and it was raised in participants who reported having ever injected with used needles and syringes. In Belgrade, anti-HCV positivity was also associated with being female, lower educational attainment, less frequent use of the same syringe before disposal, and imprisonment, whilst in Podgorica it was associated with older age, being detained or arrested by the police and having ever had a previous HCV test. These results suggest an urgent need for interventions which focus on reducing injecting risk behaviour and targeting recent initiates to injecting prior to the point of HCV transmission. Mathematical modelling studies suggest that in each population of IDUs there may be a small proportion which is at the greatest risk of acquiring HIV and HCV, and that targeting these may have a large impact on subsequent levels of infection.[35] Qualitative research in Montenegro also emphasises an adverse relationship between policing practices and injecting risk behaviour.[26] Elevated risk linked to policing and prison suggests the need for hepatitis C prevention which focuses not only on risk awareness among IDUs but on social and institutional change.[36]

### Testing

Uptake of rapid HIV testing during the study in Belgrade was relatively high, considering it was a novel intervention in this population; half of the sample wanted to know their HIV status and had pre-test counselling. This finding, similar to other evidence, suggests that rapid HIV testing in community settings can effectively target large numbers of people at high risk of HIV infection.[21] Qualitative research in Serbia suggests reluctance among IDUs to access state testing services, and a lack of normalised routine testing for anti-HIV and anti-HCV.[9,37] This is in a context of fears that confidentiality may be breached, mistrust of state health services, and a culture of non-disclosure regarding viral health status. We found complete concordance between the results from rapid testing and confirmatory testing, and no samples from HIV-infected participants registered weak positive reactions. Our rapid test algorithm therefore provided specificity comparable to EIA/Western Blot, and good sensitivity, albeit with broad confidence intervals due to few positives.

For this study each participant was tested contemporaneously by both rapid HIV POCT devices. Their accuracies were identical, albeit on a relatively small population. For routine use it is recommended that a single POCT device be used to screen individuals and that a second POCT device is held in reserve for immediate application to individuals whose initial test is reactive. Particularly in populations with a low HIV prevalence, and for which further confirmatory testing may not be available, the use of a third POCT device is recommended to minimise any risk of false positive diagnoses.[38] It is important to select POCT devices that are highly sensitive and specific, easy to perform and whose end-point reaction is easily read. Furthermore to ensure a very high positive predictive value it is necessary to demonstrate that the second (and third) POCT device is not prone to the same false positive reactions of the first. There is very little difference in performance between quality POCT devices (see http://www.who.int/diagnostics_laboratory/publications/evaluations/en/index.html for further information) and consequently the substantial additional costs of testing every individual in parallel by two devices are not counter-balanced by any worthwhile gain in sensitivity. Indeed, such approaches are likely to increase the number of indeterminate (discordant) findings, with unnecessary uncertainty for the individual and yet more expense to resolve these discrepancies.

### Limitations

A limitation of our study was that despite some piloting of the ACASI program, we only detected during analysis that one of the facilities enabling participants to skip questions was not functioning properly. This led to missing data, the potential effects of which we have attempted to minimise through the use of multiple imputation methods. Also, on occasions the numbers of IDUs trying to enrol in the study at any one time were too large to manage at the recruitment sites, with the risk that not all of those invited to return at a later appointed time actually did so. We recorded a few instances of individuals trying to enrol who did not meet the eligibility criteria for the study or attempting to enrol for a second time,(LG Johnston, personal communication) which may indicate that the incentive used in the study may have been too high.

Despite these limitations, we believe that the combination of community recruitment using RDS techniques, ACASI, and rapid HIV testing, with linked diagnostic validation, is the optimal methodology for future community surveys of IDUs. RDS provided a useful method to recruit a large number of IDUs from community settings in a relatively short space of time (six weeks in Belgrade and five weeks in Podgorica). There was strong anecdotal support among the survey staff that the study penetrated previously unknown social networks of IDUs. For instance, three weeks into the study in Podgorica groups of IDUs who were younger, more affluent and from a 'stable' area of the city, began enrolling in the study (LG Johnston, personal communication). The use of fixed sites enables survey staff to screen potential participants for eligibility to join the study, administer questionnaires by ACASI, and deliver VCT to a hard-to-reach population in an informal community setting.

## Conclusion

In settings where there are multiple social and structural barriers to gaining access to testing services,[9] rapid HIV testing may not only provide an incentive to participate in research but may also be considered an ethical public health imperative, providing research participants with low threshold access to testing services. Alongside an urgent need to provide low threshold access to testing services among IDUs who remain hidden or reluctant to contact existing medical services, our findings emphasise a general need for harm reduction interventions targeting IDUs early in their injection careers to prevent HCV and maintain containment of HIV. With only one needle and syringe outlet in each of the cities of Belgrade and Podgorica at the time of the study, and limited access to opioid substitution treatment at optimum dose, a key target is the scale-up of harm reduction services for IDUs in Serbia and Montenegro.

## Competing interests

The authors declare that they have no competing interests.

## Authors' contributions

AJ, TR, LGJ and JVP designed the study. AJ, TR, LGJ, LP, VA, DS, BM, MS and JVP contributed to fieldwork and data collection. SŽ, RPP and JVP oversaw the laboratory testing. AJ, LGJ and LP conducted the statistical analysis. All authors contributed to the drafting of the manuscript and gave final approval of the version to be published.

## Pre-publication history

The pre-publication history for this paper can be accessed here:

http://www.biomedcentral.com/1471-2334/9/14/prepub

## Supplementary Material

Additional file 1 **Table 1: Rapid testing algorithm.** This table describes the rapid testing algorithm. Click here for file

Additional file 2 **Table 2: Sample and population characteristics of injecting drug users in Belgrade and Podgorica.** This table shows the sample and population characteristics of IDUs recruited in Belgrade and Podgorica. Click here for file

Additional file 3 **Table 3: Univariable and multivariable predictors of anti-HCV positivity in Belgrade and Podgorica: logistic regression.** This table shows the univariable and multivariable predictors of anti-HCV positivity. Click here for file

Additional file 4 **Table 4: Comparison of rapid and confirmatory anti-HIV testing results.** This table shows a comparison of the results from the rapid test algorithm and the confirmatory testing. Click here for file

## References

[B1] Hamers FF Downs AM HIV in central and eastern Europe Lancet 2003 361 9362 1035 1044 10.1016/S0140-6736(03)12831-0 12660072

[B2] Rhodes T Lowndes C Judd A Mikhailova LA Sarang A Rylkov A Tichonov M Lewis K Ulyanova N Alpatova T Explosive spread and high prevalence of HIV infection among injecting drug users in Togliatti City, Russia AIDS 2002 16 13 F25 31 10.1097/00002030-200209060-00002 12218407

[B3] Rhodes T Simic M Transition and the HIV risk environment BMJ 2005 331 7510 220 223 16037463 10.1136/bmj.331.7510.220 PMC1179776

[B4] Aceijas C Rhodes T Global estimates of prevalence of HCV infection among injecting drug users Int J Drug Policy 2007 18 5 352 358 10.1016/j.drugpo.2007.04.004 17854722

[B5] Cobic P Keserovic N Radovanovic Z Dobec M Bjeljac ZS Jung M Prelic A Vukov M Baba-Milkic N Gledovic Z HIV-1 and HTLV-1 infections among intravenous drug abusers in Belgrade, Yugoslavia J Acquir Immune Defic Syndr 1990 3 12 1197 1199 2243321

[B6] Kilibarda M HIV infection among drug abusers in the Belgrade area Bulletin on Narcotics 1993 45 1 135 146 8305903

[B7] EuroHIV HIV/AIDS surveillance in Europe. End-year report 2006. No. 75 2007 Saint-Maurice: Institut de Veille Sanitaire

[B8] Rhodes T Prodanovic A Zikic B Kuneski E Pavicevic T Karadzic D Bernays S Trust, disruption and responsibility in accounts of injecting equipment sharing and hepatitis C risk Health, Risk and Society 2008 10 221 240 10.1080/13698570802160921

[B9] Rhodes T Zikic B Prodanovic A Kuneski E Bernays S Hygiene and uncertainty in qualitative accounts of hepatitis C transmission among drug injectors in Serbia Soc Sci Med 2008 66 6 1437 1447 10.1016/j.socscimed.2007.11.009 18201809

[B10] Heckathorn DD Respondent-driven sampling: A new approach to the study of hidden populations Social Problems 1997 44 2 174 199 10.1525/sp.1997.44.2.03x0221m

[B11] Erickson BH Some problems of inference from chain data Sociological Methodology 1979 10 276 302 10.2307/270774

[B12] Malekinejad M Johnston LG Kendall C Kerr LGFS Rifkin M Rutherford GW Using respondent-driven sampling methodology for HIV biological and behavioral surveillance in international settings: a systematic review AIDS Behav 12 4 Suppl S105 S130 18561018 10.1007/s10461-008-9421-1

[B13] Heckathorn DD Respondent-driven sampling II: Deriving valid population estimates from chain referral samples of hidden populations Social Problems 2002 49 1 11 34 10.1525/sp.2002.49.1.11

[B14] Salganik MJ Heckathorn D Sampling and estimation in hidden populations using respondent-driven sampling Sociological Methodology 2004 34 1 193 240 10.1111/j.0081-1750.2004.00152.x

[B15] Johnston LG Malekinejad M Kendall C Iuppa IM Rutherford GW Implementation challenges to using respondent-driven sampling methodology for HIV biological and behavioral surveillance: field experiences in international settings AIDS and Behavior 2008 12 4 Suppl S131 141 10.1007/s10461-008-9413-1 18535901

[B16] Scott G "They got their program, and I got mine": a cautionary tale concerning the ethical implications of using respondent-driven sampling to study injection drug users Int J Drug Policy 2008 19 1 42 51 10.1016/j.drugpo.2007.11.014 18226516

[B17] Sullivan PS Lansky A Drake A Failure to return for HIV test results among persons at high risk for HIV infection: results from a multistate interview project JAIDS 2004 35 5 511 518 15021316 10.1097/00126334-200404150-00009

[B18] Respess RA Rayfield MA Dondero TJ Laboratory testing and rapid HIV assays: applications for HIV surveillance in hard-to-reach populations AIDS 2001 15 Suppl 3 S49 59 10.1097/00002030-200104003-00007 11421183

[B19] Turner CF Ku L Rogers SM Lindberg LD Pleck JH Sonenstein FL Adolescent sexual behavior, drug use, and violence: increased reporting with computer survey technology Science 1998 280 5365 867 873 10.1126/science.280.5365.867 9572724

[B20] Des Jarlais DC Paone D Milliken J Turner CF Miller H Gribble J Shi Q Hagan H Friedman SR Audio-computer interviewing to measure risk behaviour for HIV among injecting drug users: a quasi-randomised trial Lancet 1999 353 9165 1657 1661 10.1016/S0140-6736(98)07026-3 10335785

[B21] Centers for Disease Control and Prevention Rapid HIV testing in outreach and other community settings – United States, 2004–2006 MMWR 2007 56 47 1233 1237 18046300

[B22] Stetler HC Granade TC Nunez CA Meza R Terrell S Amador L George JR Field evaluation of rapid HIV serologic tests for screening and confirming HIV-1 infection in Honduras AIDS 1997 11 3 369 375 10.1097/00002030-199703110-00015 9147429

[B23] San Antonio-Gaddy M Richardson-Moore A Burstein GR Newman DR Branson BM Birkhead GS Rapid HIV antibody testing in the New York State Anonymous HIV Counseling and Testing Program: experience from the field JAIDS 2006 43 4 446 450 16980908 10.1097/01.qai.0000243055.65698.51

[B24] Bucher JB Thomas KM Guzman D Riley E Dela Cruz N Bangsberg DR Community-based rapid HIV testing in homeless and marginally housed adults in San Francisco HIV Med 2007 8 1 28 31 10.1111/j.1468-1293.2007.00423.x 17305929

[B25] Viani RM Hubbard P Ruiz-Calderon J Araneta MR Lopez G Chacon-Cruz E Spector SA Performance of rapid HIV testing using Determine HIV-1/2 for the diagnosis of HIV infection during pregnancy in Tijuana, Baja California, Mexico Int J STD AIDS 2007 18 2 101 104 10.1258/095646207779949655 17331281

[B26] Gray RH Makumbi F Serwadda D Lutalo T Nalugoda F Opendi P Kigozi G Reynolds SJ Sewankambo NK Wawer MJ Limitations of rapid HIV-1 tests during screening for trials in Uganda: diagnostic test accuracy study BMJ 2007 335 7612 188 17545184 10.1136/bmj.39210.582801.BE PMC1934458

[B27] Judd A Hickman M Jones S McDonald T Parry JV Stimson GV Hall AJ Incidence of hepatitis C virus and HIV among new injecting drug users in London: prospective cohort study BMJ 2005 330 7481 24 25 15533854 10.1136/bmj.38286.841227.7C PMC539846

[B28] Judd A Parry J Hickman M McDonald T Jordan L Lewis K Contreras M Dusheiko G Foster G Gill N Evaluation of a modified commercial assay in detecting antibody to hepatitis C virus in oral fluids and dried blood spots J Med Virol 2003 71 1 49 55 10.1002/jmv.10463 12858408

[B29] Connell JA Parry JV Mortimer PP Duncan J Novel assay for the detection of immunoglobulin G antihuman immunodeficiency virus in untreated saliva and urine J Med Virol 1993 41 2 159 164 10.1002/jmv.1890410212 8283178

[B30] Tamashiro H Constantine NT Serological diagnosis of HIV infection using oral fluid samples Bull World Health Organ 1994 72 1 135 143 8131250 PMC2486497

[B31] Connell JA Parry JV Detection of anti-HIV in saliva and urine at the time of seroconversion Clin Diagn Virol 1994 1 299 311 10.1016/0928-0197(94)90060-4 15566744

[B32] World Health Organization HIV simple/rapid assays: operational characteristics (Phase 1). Report 12 whole blood specimens 2002 Geneva: World Health Organization

[B33] Heckathorn DD Semaan S Broadhead RS Hughes JJ Extensions of respondent-driven sampling: a new approach to the study of injection drug users aged 18–25 AIDS and Behavior 2002 6 1 55 67 10.1023/A:1014528612685

[B34] Horton NJ Kleinman KP Much ado about nothing: A comparison of missing data methods and software to fit incomplete data regression models The American Statistician 2007 61 1 79 90 17401454 10.1198/000313007X172556 PMC1839993

[B35] Sutton AJ Hope VD Mathei C Mravcik V Sebakova H Vallejo F Suligoi B Brugal MT Ncube F Wiessing L A comparison between the force of infection estimates for blood-borne viruses in injecting drug user populations across the European Union: a modelling study J Viral Hepat 2008 15 11 809 816 18761605 10.1111/j.1365-2893.2008.01041.x

[B36] Rhodes T Treloar C The social production of hepatitis C risk among injecting drug users: a qualitative synthesis Addiction 2008 103 10 1593 1603 10.1111/j.1360-0443.2008.02306.x 18821870

[B37] Rhodes T Prodanovic A Zikic B Kuneski E Bernays S Trust, disruption and responsibility in accounts of injecting equipment sharing and hepatitis C risk in Serbia and Montenegro Health, Risk and Society in press

[B38] Joint United Nations Programme on HIV/AIDS (UNAIDS) – WHO Revised recommendations for the selection and use of HIV antibody tests WER 1997 72 81 88 9238418

